# Thyroid disorders in Brazil: time for action: Distúrbios da tireoide no Brasil: hora de agir

**DOI:** 10.1590/1516-3180.2016.1344040716

**Published:** 2015-11-13

**Authors:** Paulo Andrade Lotufo

**Affiliations:** 1 MD, DrPH. Full Professor, Department of Internal Medicine, Faculdade de Medicina da Universidade de São Paulo (FMUSP), São Paulo, SP, Brazil.

Chronic diseases are the leading cause of death and hospitalization in Brazil. In this regard, attention has been drawn to cardiovascular, neoplastic, respiratory, digestive and mental diseases, but not to thyroid disorders.^1^^,^^2^ There is certainly a contradiction between the epidemiological profile and the fact that thyroid conditions are one of the most prominent points of interest for clinicians. This discrepancy between the collective and individual approach probably arises because diagnosis, treatment, screening and prevention of thyroid diseases have been outstanding actions of both public health and medicine over the last two centuries. Nowadays, occurrences of patients with myxedematous facies and Graves's disorder are less frequent because of the combination of awareness among physicians of the symptoms and signs of thyroid dysfunction, availability of thyroid tests and use of inexpensive medicines for thyroid replacement or for blocking hyperfunction of the thyroid gland.

One successful public health action that is rarely mentioned by epidemiologists is the iodine supplementation that was proposed 170 years ago, which may have been one of the first examples of "translational medicine." Goiter has been treated in China with marine sponges and seaweeds since 1900 BC and in Europe with burnt and dried marine sponges, cuttlefish and starfish since the 13^th^ century. In 1812, Gay-Lussac isolated the chemical element iodine from seaweed. Very soon thereafter, in 1813, physicians in Switzerland and England hypothesized that iodine should be the first-choice treatment for people with goiter, and iodine has been widely prescribed since then for people with goiter. In Colombia in 1825, J. Baptiste Boussingault recommended goiter prophylaxis with iodized salt.^3^

It may be that iodine deficiency after the postnatal period is a cause of goiter: in fact, a compensatory disease. Furthermore, iodine deficiency in utero can impair brain development, with marked effects on the psychoneural evolution of newborns, children and adolescents, thus leading to adults with an intellectual handicap called cretinism. Screening for hypothyroidism was applied worldwide subsequent to the results from the New England Congenital Hypothyroidism collaborative study, in which newborns with hypothyroidism received treatment from the 5^th^ to the 40^th^ day of life, i.e. before the appearance of any recognizable clinical manifestations, which led to good intelligence quotients.^4^

Today, there is serious concern about how effective the neonatal screening and iodine supplementation polices implemented in Brazil have been. This is due not only to the impact on children's intellectual development caused by lack of access to screening, but also to the effects of under or oversupplementation of iodine. High levels of salt iodination can possibly be correlated with outcomes such as thyroid cancer and imbalance of thyroid hormone distribution in the population, thereby elevating the proportion of people with subclinical hypothyroidism or hyperthyroidism. Unfortunately, two initiatives funded by the Ministry of Health, named the National Program for Neonatal Screening (NPNS) and the National Survey for Evaluation of the Impact of Salt Iodation (Pesquisa Nacional para Avaliação do Impacto da Iodação do Sal, PNAISAL), have not released updated data coming from the results from these surveys.

## Neonatal screening for hypothyroidism

In Brazil, neonatal screening for congenital hypothyroidism began in 1976, through voluntary work by Dr. Benjamin Schmidt, a professor of Pediatrics, at APAE-SP (a non-governmental organization of parents and friends of disabled children in the state of São Paulo). Only in 2001 was the formal NPNS established, organized in all states. To the best of our knowledge, no recent data evaluating this program is available, except for one paper authored by Nascimento, who analyzed data from the NPNS and found that there were huge differences in screening performance among the Brazilian states. The coverage of the NPNS system was 82% among newborns, but blood samples were only collected within the first seven days of life for 57% of them. Moreover, a delay between arrival of the blood at the laboratory and release of the results was observed.^5^

In conclusion, to the best of our knowledge, there is no recent national data on the status of neonatal screening for hypothyroidism, and the old news is not exactly good news.

## Salt iodination

In Brazil, salt iodination was established in the 1950s, but the amount of iodine added to salt has varied since then. Over the last 30 years, addition of different amounts of iodine (in milligrams) per kilogram of salt has been stipulated: 10-30 (1984); 40-60 (1994); 40-100 (1999); 20-60 (2003); and 15-45 (2013). Before this apparent mess of state-driven guidelines, three national surveys among schoolchildren showed a downward trend of goiter prevalence rates: 25% (1955), 15% (1975) and a median of 1.3% (1996).^6^

Today, the data on iodine supplementation in Brazil are divergent. A national survey called PNAISAL on 19,600 children aged 6 to 14 years began in 2008 and ended in 2011.^7^ However, the results have so far not been made available. Meanwhile, there have been other surveys in several locations indicating lower or higher levels of urinary iodine and differences in the prevalence of goiter.^8^^,^^9^^,^^10^

In conclusion, to the best of our knowledge, no recent national data on the prevalence of goiter or on urinary iodine levels is available to enable rational actions towards thyroid health.

## Thyroid cancer

In 2013, the incidence of thyroid cancer for both sexes ranked 19^th^ worldwide and 9^th^ in Brazil, among types of cancer. In contrast, the death rates relating to thyroid cancer were similar worldwide and in Brazil, respectively the 25^th^ and 23^rd^ most lethal malignant neoplasm.^11^ The initial conclusion from these data could be that overdiagnosis is occurring in Brazil. Nevertheless, comparison between the cancer registry of Sao Paulo and the United States (US) National Cancer Institute's Surveillance Epidemiology End Results (SEER) revealed that there was higher incidence of thyroid cancer in São Paulo than in the US. The higher rate of thyroid cancer was differential, such that there were more cases of the papillary type than of the follicular type. The higher papillary-to-follicular ratio observed in São Paulo than in the US weakens the medical surveillance hypothesis and strengthens another hypothesis relating to excess iodination, which is a risk factor for papillary but not for follicular thyroid cancer.^12^

In conclusion, differences in iodine nutrition status between São Paulo and the US SEER population may have affected the incidence patterns observed.

## Subclinical thyroid disorders

The concept of subclinical disorders is familiar, but it is in fact weird. To understand the meaning of "subclinical disorders," we must look back to a historical debate on the definition of hypertension that was conducted in the 1950s. There were two sides. On one side, led by Richard Platt, hypertension was defined as a disease that could be present or absent. In opposition to this, George Pickering stated the view that now prevails: hypertension is only an arbitrary point on a continuous curve of blood pressure risk.^13^

During the 1980s, myxedema coma and thyroid storm were still being diagnosed in Brazil. However, because of improvements in laboratory tests and medical access, clinical cases of hypo and hyperthyroidism are diagnosed very early in the clinical phase of the disease. Individuals with subclinical disorders of the thyroid have a condition without a clinical picture but with a higher risk of evolving into overt disease than what is seen among individuals who have healthy thyroid function. There are data on the Brazilian population from two studies.

The first is the "São Paulo Ageing & Health Study (SPAH)", which studied a population-based sample of low-income elderly people greater than or equal to 65 years of age and revealed that presence of subclinical hyperthyroidism showed a fourfold greater association with the presence of any dementia or vascular dementia.^14^^,^^15^ The other is the "Brazilian Longitudinal Study of Adult Health" (ELSA-Brasil), a multicenter cohort study on 15,105 public employees (35-74 years of age) in six Brazilian cities.^16^ ELSA-Brasil showed that participants with subclinical hyperthyroidism had a 2.5 times greater chance of presenting panic disorder, while those who had subclinical hypothyroidism had a 25% lower frequency of generalized anxiety disorder.^17^ Moreover, ELSA-Brasil showed that thyrotropin levels had associations with insulin resistance and subclinical atherosclerosis.^18^^,^^19^

In conclusion, the effect of thyroid hormones should be analyzed as a risk factor for adverse outcomes regarding cardiovascular and mental functions.

## Final remarks

This editorial presents many more questions than answers about thyroid epidemiology in Brazil. It recognizes the important role that thyroidologists, dietitians and public health specialists working under harsh conditions have played over many decades, in creating and maintaining programs relating to neonatal screening and salt iodination. However, it is undeniable that these programs deserve a place within the continuous processes of research policy. Continuing collaboration among all individuals with an interest in this fascinating chapter of the biological and health sciences needs to be invigorated by the Ministry of Health. Lastly, this Journal is opening its pages for new insights on thyroid diseases in Brazil.
